# Temperature × light interaction and tolerance of high water temperature in the planktonic freshwater flagellates *Cryptomonas* (Cryptophyceae) and *Dinobryon* (Chrysophyceae)

**DOI:** 10.1111/jpy.12826

**Published:** 2019-01-31

**Authors:** Christina Wirth, Romana Limberger, Thomas Weisse

**Affiliations:** ^1^ Research Department for Limnology University of Innsbruck Mondseestr. 9 5310 Mondsee Austria

**Keywords:** cell size, *Cryptomonas*, *Dinobryon*, food webs, lake warming, phytoplankton, temperature × light interaction, temperature tolerance

## Abstract

Using microcosm experiments, we investigated the interactive effects of temperature and light on specific growth rates of three species each of the phytoplanktonic genera *Cryptomonas* and *Dinobryon*. Several species of these genera play important roles in the food web of lakes and seem to be sensitive to high water temperature. We measured growth rates at three to four photon flux densities ranging from 10 to 240 μmol photon · m^−2^ · s^−1^ and at 4–5 temperatures ranging from 10°C to 28°C. The temperature × light interaction was generally strong, species specific, and also genus specific. Five of the six species studied tolerated 25°C when light availability was high; however, low light reduced tolerance of high temperatures. Growth rates of all six species were unaffected by temperature in the 10°C–15°C range at light levels ≤50 μmol photon · m^−2^ · s^−1^. At high light, growth rates of *Cryptomonas* spp. increased with temperature until the temperature optimum was reached and then declined. The *Dinobryon* species were less sensitive than *Cryptomonas* spp. to photon flux densities of 40 μmol photon · m^−2^ · s^−1^ and 200 μmol photon · m^−2^ · s^−1^ over the entire temperature range but did not grow under a combination of very low light (10 μmol photon · m^−2^ · s^−1^) and high temperature (≥20°C). Among the three *Cryptomonas* species, cell volume declined with temperature and the maximum temperature tolerated was negatively related to cell size. Since *Cryptomonas* is important food for microzooplankton, these trends may affect the pelagic carbon flow if lake warming continues.

**Abbreviations**
B: biomass
BP: specific biomass production rate
N: cell number
*T*_max_: maximum temperature tolerated
*T*_opt_: temperature optimum
V: cell volume
μ_max_: highest growth rate observed or predicted
μ: specific growth rate

Phytoplankton is of the utmost importance for aquatic ecosystem processes and global biogeochemical cycling, providing half of the primary production on earth (Field et al. 1998, Boyce et al. 2010). Phytoplankton growth is primarily controlled by light, nutrient supply, water temperature, and the interactions between these (reviewed by Padisák 2003, Reynolds 2006). It is often difficult to decipher the antagonistic effects of simultaneously acting bottom‐up versus top‐down forces (via predation and parasitism) on phytoplankton biomass from field data. Monoculture experiments under well‐controlled conditions are crucial to provide a mechanistic understanding of phytoplankton growth in response to the major environmental drivers. Such experiments often focus on the independent effects of environmental variables. However, environmental drivers interact in their effects on phytoplankton growth (i.e., the effect of one variable is dependent on the states of other variables in the system; Frossard et al. 2018).

The independent effects of each of the above variables have been studied with an array of different algal species. Temperature affects the metabolic processes of all organisms and, therefore, strongly affects algal replication rates. The effects of nutrient supply on algal growth have been investigated in detail, namely in freshwater species, in the decades following Hutchinson's seminal article on the “Paradox of the plankton” (Hutchinson 1961), paving the way for Tilman's “mechanistic theory of resource competition” (Tilman 1981, 1982). Similarly, the growth rate and photosynthesis versus irradiance relationships (P‐I curve) have been investigated for many marine and freshwater species (reviewed by Padisák 2003, Reynolds 2006). Taken together, the theory of resource‐based competition provided a framework to explain “bottom‐up” control and seasonal succession of major phytoplankton taxa (e.g., diatoms, cryptophytes, dinoflagellates) in many lakes. However, in most cases it is not possible to predict the occurrence and abundance of individual species in a multi‐species environment (Huisman and Weissing 2001). This is because competition for three or more resources may generate oscillations and chaotic fluctuations in species abundances (Huisman and Weissing 1999).

Concerning the interactive effects of several variables, it is undisputed that the effect of temperature on phytoplankton growth depends on resource availability. Edwards et al. (2016) investigated the interactive effects of light and temperature on phytoplankton growth based upon published experiments on 57 phytoplankton species. Light‐limited growth, light‐saturated growth, and the optimal irradiance for growth were all highly sensitive to temperature. However, the maximum achievable growth rate (μ_max_) was invariant with temperature under strong light limitation. Edwards et al. (2016) also found that light limitation reduces a species’ thermal optimum by ~5°C.

The above conclusions (Edwards et al. 2016) were reached by a meta‐analysis across phylogenetically and physiologically different taxa. It is generally assumed that closely related taxa respond similarly to environmental conditions. For instance, the current conjecture is that lake warming generally benefits cyanobacteria (Moss et al. 2011, Paerl and Paul 2012, Bergkemper and Weisse 2017). However, various colonial and filamentous cyanobacteria belong to different functional groups (Reynolds et al. 2002, Reynolds 2006), i.e., they are not functionally redundant. Functional redundancy or ecological equivalence implies that several or even many functionally similar species provide biological buffering capacity, allowing relatively stable community functions (e.g., primary and secondary production, nutrient cycling) in spite of taxonomic changes (reviewed by Weisse 2017). It is an open question if all species within seemingly homogenous taxa such as the cryptophyte genus *Cryptomonas* (group Y in Reynolds et al. 2002) or the chrysophyte genus *Dinobryon* (group E in Reynolds et al. 2002) respond similarly to environmental change. For instance, cell size is highly variable in *Cryptomonas* spp. (Morgan and Kalff 1979, Ojala 1993a, Montagnes et al. 2008). However, it is unknown if cell volumes of small and large species within this genus respond similarly to temperature × light interaction.

Cell size is a key trait of planktonic protists (Weisse 2017). Temperature has been found to reduce phytoplankton cell size (Daufresne et al. 2009, Yvon‐Durocher et al. 2011), both by shifting community composition to smaller taxa and populations to smaller individuals. The species‐shift hypothesis (Daufresne et al. 2009, Sommer et al. 2017) predicts that the proportion of small‐sized species generally increases with temperature in the course of global warming, with important implications for the planktonic food web (Bergkemper et al. 2018). This is mainly because zooplankton grazing on algae is size selective, and smaller cells have a higher surface‐to‐volume ratio than larger cells (Reynolds 2006), thereby favoring resource use (i.e., of light, nutrients) and, in consequence, yielding higher specific growth rates. Therefore, shifts in species composition and average cell size may lead to altered ecosystem functioning.

Temperate lakes are currently warming up rapidly, with global mean lake summer surface water temperature (SWT) increasing at 0.34°C per decade (O'Reilly et al. 2015). Similarly, the maximum water temperature recorded in each year has increased significantly in many lakes (Dokulil 2018). However, climate change can also influence phytoplankton abundance and composition by altering resource availability. For example, the earlier onset and later breakdown of thermal stratification increases light availability, while increased runoff from the catchment during periods of heavy precipitation or snowmelt reduces light availability (Winder and Sommer 2012, Bergkemper and Weisse 2017). Heat waves, which are currently becoming more frequent in many temperate areas (IPCC 2013), are usually coupled to high irradiances and low nutrient load because of reduced runoff. The question arises if climatic change causes major shifts in the taxonomic composition of the phytoplankton assemblage, favoring heat‐tolerant taxa such as cyanobacteria at the expense of eukaryotic species whose occurrence is constrained by high water temperature (Moss et al. 2003, Bergkemper and Weisse 2017).

The present study focuses on the interactive effects of light and temperature on specific growth rates of two freshwater algal genera. Species of the cryptophyte genus *Cryptomonas* and the chrysophyte genus *Dinobryon* are common constituents of freshwater phytoplankton and both might respond negatively to lake warming. Several experimental studies demonstrated that species of these two genera might be sensitive to high water temperature (≥25°C; Butterwick et al. 2005, Heinze et al. 2013, Weisse et al. 2016, Bergkemper et al. 2018). These findings are supported by field data. Based upon detailed data sets from mesotrophic Lake Lacawac (Pennsylvania, USA) and several other lakes in North America and Europe, Heinze et al. (2013) concluded that high abundances of *Dinobryon* spp. were associated with a narrow range of temperature (9°C–18°C). The maximum temperature at which *Dinobryon* species were present did not exceed 26°C. Similarly, *Cryptomonas* spp. typically peak in spring and early summer in deep temperate lakes (Sommer 1986, Sommer et al. 1986, Reynolds 2006). Some Arctic and subarctic lakes can be characterized as Chrysophycea‐Cryptophyceae lakes (Holmgren 1983, reviewed by Reynolds 2006). Recently, Bergkemper and Weisse (2017) noted that *Cryptomonas* spp. declined in the epilimnion of an oligo‐mesotrophic Austrian lake in the course of a heat wave when water temperature reached 22°C–27°C.

Accordingly, although exceptions of heat‐tolerant species have been reported both among *Cryptomonas* spp. (Cloern 1977, Ojala 1993b) and *Dinobryon* spp. (Butterwick et al. 2005), our main hypotheses were that (i) typical members of both genera would not tolerate temperatures ≥25°C over several generations, (ii) the effect of temperature on the specific growth rate would depend on light intensity, and (iii) the independent and interactive effects of temperature and light would differ between species and genera. More precisely, we expected that light limitation would increase sensitivity to high temperatures and that the mixotrophic species of the genus *Dinobryon* would be less sensitive to low light levels than the *Cryptomonas* species.

We tested our hypotheses with three common species of each genus in laboratory microcosm experiments. If our assumptions were correct, implications for the planktonic food web would be evident, mainly at high water temperatures. First, this is because *Cryptomonas* belongs to the preferred food of many protist and microcrustacean grazers in many lakes (Dokulil 1988, Klaveness 1988/1989, Reynolds 2006, Posch et al. 2015). However, its significance in the food web may be sensitive to temperature. Modelling results derived from microcosm experiments with the same *Cryptomonas* sp. strain as used in the present study as food and a common ciliate as predator demonstrated that carbon production of both prey and predator peaked at 20°C and rapidly declined above this maximum (Montagnes et al. 2008). Notably, cell volume of *Cryptomonas* sp. decreased linearly with temperature ranging from 8°C to 27°C in the previous study (Montagnes et al. 2008). Accordingly, we expected that cell volumes of all three *Cryptomonas* species used in the present investigation would decline with temperature. Secondly, *Dinobryon* plays an important role as consumer of bacteria (Bird and Kalff 1986, 1987), and bacterial uptake is relatively more important at low than at high irradiances (Princiotta et al. 2016). These authors reported that in *D. sociale*, bacterial ingestion and photosynthesis increased with temperature to a maximum at 16°C, and declined at 20°C. Therefore, if our above postulates were valid, the relative significance of *Cryptomonas* and *Dinobryon* in the planktonic food web would decrease if lake warming continues.

## Materials and Methods

### Study organisms

The *Cryptomonas* species investigated were obtained from either the Culture Collection of Algae and Protozoa (*C. curvata*, strain CCAP 979/62) or the Culture Collection of Algae at Goettingen University (SAG, *Cryptomonas* sp. strain SAG 26.80 and *C. pyrenoidifera* strain SAG 979‐3). The latter strain was originally designated *C. ovata* var. *palustris* but later identified as *C. pyrenoidifera* (Hoef‐Emden and Melkonian 2003). In contrast with the other two species, *Cryptomonas* sp. strain SAG 26.80, which had been isolated from Lake Windermere (UK), has not yet been described in detail. However, this strain has been used frequently as food for various protist and metazoan predators (Weisse and Kirchhoff 1997, Müller and Schlegel 1999, Weisse and Frahm 2002, Montagnes et al. 2008) and its SSU rDNA and several other genes have recently been sequenced (T. Pröschold, pers. comm.). The three *Dinobryon* species studied were isolated by one of us (RL) from Lake Lucerne in early August, 2017. *Dinobryon divergens* and *D. sociale* were taken from 5 m depth, *D. sertularia* from the surface. All algal cultures were non‐axenic monocultures.

### Stock cultures

All *Cryptomonas* stock cultures were maintained in WC medium modified according to the recipe of the *Culture collection of Algae and Protozoa* (www.ccap.ac.uk). The strains were kept in culturing flasks (CytoOne^®^, 50–250 mL volume) at 15°C ± 1°C under a photoperiod of 16:8 h light:dark (L:D). Photon flux density (tantamount to “light intensity” and “irradiance” in the following) during the light phase was ~200 μmol photon · m^−2^ · s^−1^.

The three *Dinobryon* strains were cultivated in similar flasks with DY‐Vm medium prepared according to the recipe of the *National Center for Marine Algae and Microbiota* (www.ncma.bigelow.org), diluted 1:1 with sterile filtered water from Lake Mondsee, at 15°C ± 1°C and 16:8 h L:D. Light intensity during the light period was 100 μmol photon · m^−2^ · s^−1^.

### Experimental design

The experiments were conducted at 4–5 temperatures ranging from 10°C to 25°C, respectively, the maximum temperature tolerated (*T*_max_) by a given species, and three different photon flux densities. All treatment combinations were replicated three times. We used incubators (Model KBK/LS 4330; Ehret, Freiburg, Germany) equipped with 16 LED‐Lights (TIROLED, type no. 1036140) and temperature control to maintain the experimental target levels. Because we had only two incubators with identical light regime available, we were not able to compare all temperature levels simultaneously.

All *Cryptomonas* spp. experiments were conducted in tissue culture flasks of 75 mL volume (CytoOne^®^, CC7682‐4825; STARLAB, Milton Keynes, UK) with vented filter caps, filled with 50 mL medium. Experimental cultures were inoculated from exponentially growing stock cultures and gradually acclimated to the experimental light and temperature conditions; temperature was changed by 0.5–1.0°C · d^−1^. At the end of the acclimation phase, the cultures were diluted with sterile filtered water from Lake Mondsee to yield an initial abundance of ~10,000 cells · mL^−1^ at temperatures ≤20°C, respectively, ~20,000 cells · mL^−1^ at temperatures >20°C. We used a higher starting density at high temperatures to obtain statistically reliable results in case that some strains would rapidly decline.

Each experiment was performed at three different light levels (50 μmol photon · m^−2^ · s^−1^, 100 μmol photon · m^−2^ · s^−1^ and 200 μmol photon · m^−2^ · s^−1^). For *Cryptomonas* sp. and *C. pyrenoidifera*, we also measured the growth response at 25°C and 240 μmol photon · m^−2^ · s^−1^. The low and medium light intensities were manipulated by wrapping the flasks with woman's tights (SILCADA classic, Esda Feinsstrumpffabrik, Oberlungwitz, Germany; Bergkemper et al. 2018). Light intensities in the incubator were measured by a spherical quantum sensor (Model SPQA 3818; LI‐COR Biosciences, Lincoln, NE, USA). A 2π‐sensor (Model LI‐1400; LI‐COR Biosciences) was used to measure light levels inside of the flasks. Both sensors measure photosynthetically active radiation (PAR, 400–700 nm, in μmol photon · m^−2^ · s^−1^). All cultures were kept for at least 24 h at the final experimental conditions prior to the beginning of the experiments. The culture flasks were rotated randomly and shaken every 24 h to guarantee equal conditions during the experiments (Bergkemper et al. 2018). Experimental duration ranged from 3 to 5 d. We did not measure pH in the course of the experiments because similar previous work in our laboratory had shown that changes in pH levels in experimental containers were minor and almost constant in each replicate over a period of 3 weeks (Weisse et al. 2016).

In the experiments with *Dinobryon* spp. we measured growth rates at four temperatures (10°C, 15°C, 20°C, 25°C) and three light intensities (10, 40, 200 μmol photon · m^−2^ · s^−1^). Because we wanted to measure growth rate at light limitation and expected less sensitivity to low light in *Dinobryon* than in *Cryptomonas*, we used lower light intensities than in the *Cryptomonas* experiments. To manipulate light intensity, we wrapped 75‐mL culture flasks with neutral density filters (Solar Graphics, Clearwater, FL, USA) and reduced irradiance to 40 μmol photon · m^−2^ · s^−1^ (moderate light) and 10 μmol photon · m^−2^ · s^−1^ (low light). At temperatures ranging from 10°C to 20°C, we acclimated the cultures to the experimental conditions for 7 d each. In the experiments at 25°C, we increased the temperature stepwise from 23°C to 25°C and then kept the experimental flasks for 3 d before the beginning of the experiments. Depending on the specific growth rates, the acclimation phase spanned from a few to ~7 generations.

At the start of the experiments, 40 mL of medium were inoculated with 1.2 mL of acclimated culture. The flasks were sampled daily over a period of 9 d. Due to the daily subsampling (see below), the liquid volume in the flasks was reduced and the gaseous volume increased in the course of the experiments, rendering it unlikely that dissolved inorganic carbon limitation affected the experimental results. In addition, the use of culture flasks with vented caps permitted gas exchange with the atmosphere.

Since *Dinobryon* species are mixotrophic, we provided a similar, potentially saturating bacterial biomass to all experimental treatments. To avoid that possible differences in associated bacteria between the three species influenced the results of the experiment, we homogenized the bacterial community at the start of the acclimation phase and again at the start of the experiment. To this end, we filtered a mixture of all cultures through a Nuclepore filter (1‐μm Whatman Nuclepore, Track Etch Membrane) and added 3 mL of the bacterial filtrate to each experimental flask.

### Sampling and analyses

Cell numbers of *Cryptomonas* spp. were measured by acoustic flow cytometry (Attune™ NxT Acoustic Focusing Cytometer, Life Technologies, Austria, Thermo Fisher Scientific Inc., Vienna, Austria; Bergkemper and Weisse 2017, Weisse and Bergkemper 2018) from 3‐mL live samples taken daily during the experiments. We used the instruments settings reported in Bergkemper and Weisse 2017). Cell size was analyzed from the same samples using FlowCAM (Flow Cytometer and Microscope; Fluid Imaging Technology, Yarmouth, ME, USA; Bergkemper and Weisse 2017, 2018) equipped with a 80 μm‐wide FC80FV flow cell and a 10× objective. Measurements were conducted in trigger mode at a flow rate of 0.15 mL · min^−1^, using the green laser installed (532 nm) to excite cellular Chl‐*a* and accessory pigments such as phycoerythrin. Classification of all samples was conducted using the Visual Spreadsheet 3.7.5 software. *Cryptomonas* cell volume was calculated from lengths and widths measurements assuming the shape of a prolate spheroid (Hillebrand et al. 1999).

In the experiments with *Dinobryon* spp., we did not use the semi‐automated optical methods for determination of their cell numbers because (i) flow cytometry measures particle concentration, not cell concentration, and therefore underestimates cellular abundance of colonial species (Weisse and Bergkemper 2018); (ii) large *Dinobryon* colonies may clog the 80‐μm wide flow cell that we used with our FlowCAM instrument. Therefore, we measured *Dinobryon* spp. abundances microscopically in Lugol's‐fixed samples taken each day or every second day in the course of the experiments. Counting was done in multiwell plates (CytoOne^®^; Starlab GmbH, Hamburg, Germany) using inverted microscopy (Nikon Eclipse TS100; Nikon CEE GmbH, Vienna, Austria) at 100× magnification. For each culture, we counted samples from four sampling dates during the exponential growth phase.

To check if bacteria limited growth of *Dinobryon*, we measured bacterial cell numbers in a number of selected samples. We quantified abundances of bacteria by flow cytometry in formaldehyde‐fixed samples after staining with the fluorochrome Syto13 (Molecular Probes, Eugene, OR, USA; Gasol and del Giorgio 2000). Uptake of bacteria‐sized latex beads and fluorescently labelled bacteria has also been demonstrated for several *Cryptomonas* species (Porter 1988, Sinistro et al. 2006 and references therein) but bacterial ingestion among cryptophytes is mainly restricted to non‐pigmented forms (Grujcic et al. 2018). Weisse et al. (2016) found no indication that the same *Cryptomonas* sp. strain as used in the present investigation ingested picocyanobacteria. Therefore, we did not measure bacterial levels in the experiments with *Cryptomonas* spp.

Specific algal growth rates (μ, d^−1^) were calculated from the slope of the linear regression of ln cell numbers (*N*) versus time (*t*) in each experiment. Specific biomass production rates (BP, d^−1^) were estimated from changes in biomass (*B*) over time:

$$\text{BP}\;\left(d^{-1}\right)=\ln\left(\frac{B_{t}}{B_{0}}\right)*\frac{1}{t}$$

where *B* is the product of *N* and the corresponding average cell volume (*V*), which was measured upon each sampling. The slope of the regression of ln *B* versus time yields BP. For simplicity, BP will be referred to as production rate in the following; BP was calculated only for *Cryptomonas* spp. because cell volume of *Cryptomonas* species are highly variable, depending on growth conditions (Morgan and Kalff 1979, Dokulil 1988, Montagnes et al. 2008) and may vary even in exponentially growing cultures under standard conditions by 50% (Weisse et al. 2001).

### Statistical analyses

The interactive effects of temperature, light and species were tested by three‐way ANOVA. Within each species, temperature × light interaction was investigated with two‐way ANOVA. The ANOVA assumptions were checked using Shapiro‐Wilk and Brown‐Forsythe tests. The Holm‐Sidak post hoc test was used for multiple pairwise comparisons of variables. Results of all tests were considered significant if *P* < 0.05. All statistical analyses were computed with the statistics package in SigmaPlot version 14.0 (Systat Software GmbH, Erkrath, Germany).

## Results

### Cell volume, growth, and production rates of *Cryptomonas* spp. in response to light and temperature

**Figure 1:**
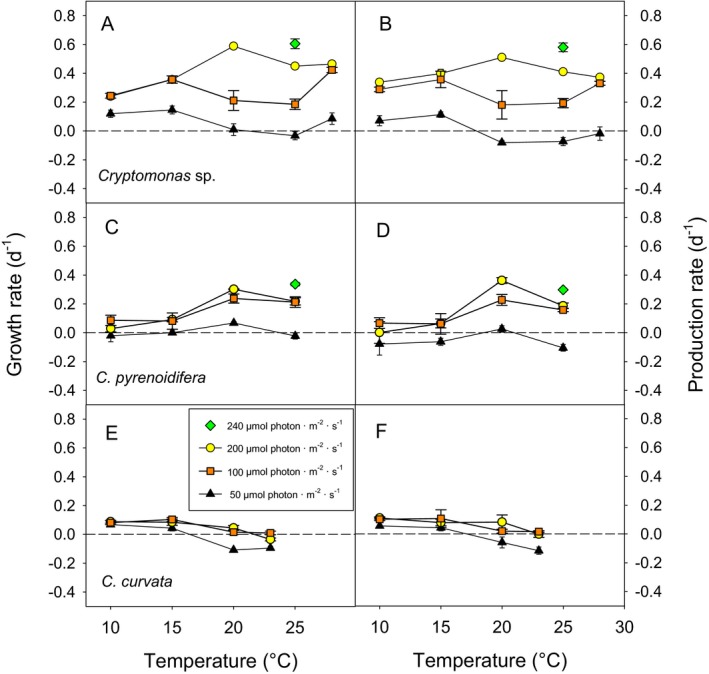
Average growth and production rates of *Cryptomonas* spp. at three photon flux densities. Error bars denote 1 standard deviation (SD).

Light intensity, temperature, and light × temperature interaction significantly affected growth and production rates of each *Cryptomonas* species (Table S1 in the Supporting Information), and the growth response in relation to light and temperature was species‐specific (Fig. 1, Table 1). Light levels positively affected temperature tolerance of the three *Cryptomonas* species. The factor “species” significantly interacted with light, temperature and their interaction (light × temperature × species).

**Table 1:** Three‐way ANOVA results for growth and production rates of *Cryptomonas* spp. with *F*‐values and significance levels (*P*); df, factor degrees of freedom

Variable(s)	df	*F*	*P*
Growth rate (μ, d^−1^)			
Temperature	2	23.58	<0.001
Species	2	433.03	<0.001
Light	2	277.92	<0.001
Temperature × species	4	72.64	<0.001
Temperature × light	4	62.57	<0.001
Species × light	4	47.34	<0.001
Temperature × species × light	8	15.10	<0.001
Production rate (BP, d^−1^)			
Temperature	2	4.84	0.012
Species	2	159.05	<0.001
Light	2	188.05	<0.001
Temperature × species	4	40.54	<0.001
Temperature × light	4	22.53	<0.001
Species × light	4	31.68	<0.001
Temperature × species × light	8	2.62	0.017

In the experiments with three irradiance levels, highest growth rates (μ_max_ = 0.59 · d^−1^) and production rates (BP = 0.51 · d^−1^) were recorded for *Cryptomonas* sp. at 20°C and the highest light intensity (Fig. 1, A and B). The peak was shifted to 25°C when the algae were exposed to 240 μmol photon · m^−2^ · s^−1^ (BP = 0.58 · d^−1^). At low light, growth and production rates of this strain were generally low and close to zero or negative at 20°C–28°C.

The shift in μ_max_ with light intensity recorded for *Cryptomonas* sp. did not occur in *C. pyrenoidifera*. Specific growth rates were not significantly higher (*P* = 0.227; *t*_4_ = −1.426) at 25°C and 240 μmol photon · m^−2^ · s^−1^ than at 20°C and 200 μmol photon · m^−2^ · s^−1^ (Fig. 1, C and D). Since the average cell volume was relatively small at 240 μmol photon · m^−2^ · s^−1^ (800–900 μm^3^), BP was lower at 25°C than at 20°C (Fig. 1D). Accordingly, irrespective of light intensity, production rates of *C. pyrenoidifera* increased with temperature between 10°C and 20°C and then declined at 25°C. Under the lowest light intensity, *C. pyrenoidifera* reached positive growth rates only at 20°C. Growth and production rates were lowest for *C. curvata* and declined with temperature >15°C (Fig. 1, E and F). This strain reached optimum conditions at 10°C–15°C under moderate to high light levels (Fig. 1, E and F); *C. curvata* was confined to 10°C–23°C and needed light levels ≥100 to thrive at temperatures ≥20°C.

Treatment effects on production rates were similar to effects on growth rates (Fig. 1), because relative changes of cell numbers and cell size in response to temperature and light were similar in the course of the experiments. Overall, cell volume declined significantly with decreasing light intensity and increasing temperature (Figs. S1–[Link], [Link] in the Supporting Information). This was most obvious in *Cryptomonas* sp. with final experimental cell volume at the highest light intensity declining from 605 μm^3^ to 260 μm^3^ over the temperature range from 10°C to 28°C (Fig. S1). Only at the maximum temperature, cell volume did not differ between the three light levels (Fig. S1E). In *Cryptomonas pyrenoidifera*, cell volume (ranging from 590 to 1,220 μm^3^) declined from 10°C to 20°C and was similar at 25°C (Fig. S2). In the largest *Cryptomonas* species, *C. curvata*, cell volume was least affected by changing light and temperature conditions, ranging from 4,600 to 6,700 μm^3^ in the various treatments (Fig. S3).

### Specific growth rates of *Dinobryon* spp. in response to light and temperature

**Figure 2:**
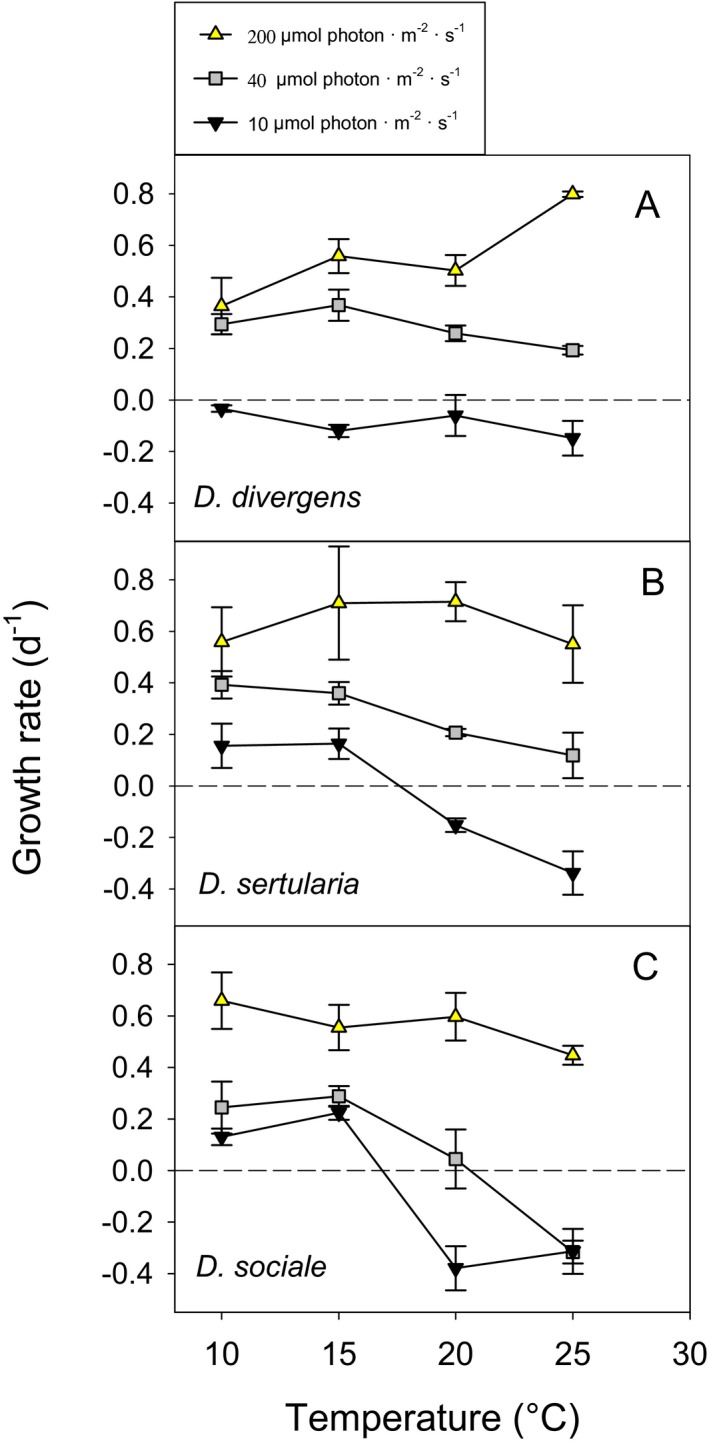
Average growth rates of *Dinobryon* spp. at three photon flux densities. Error bars denote 1 standard deviation (SD).

Similar to *Cryptomonas* spp., specific growth rates of the three *Dinobryon* species investigated depended on light level, temperature, species, and their interactions (Table 2). Separate analyses for the three species revealed significant effects of light, temperature and their interaction for each species (Table S2 in the Supporting Information). In all three species, lower light levels resulted in lower growth rates, and the relative reduction in growth rates from high to low light levels increased with temperature (Fig. 2). Sensitivity to very low light varied among species: *D. divergens* was unable to grow at 10 μmol photon · m^−2^ · s^−1^, irrespective of temperature, while *D. sertularia* and *D. sociale* grew at very low light when temperature was 10°C or 15°C. The effect of temperature varied among species and depended on light intensity (Table 2, Fig. 2). All three species were able to grow over the entire temperature range when light intensity was high, while none of the species was able to grow at temperatures ≥20°C when light intensity was very low, i.e., lower light levels resulted in reduced tolerance of high temperature. At the highest light intensity tested, μ_max_ ranged from 0.65 to 0.80 per day and was reached at different temperatures, i.e., 25°C in *D. divergens* (Fig. 2A) and 10°C in *D. sociale* (Fig. 2C). In *D. sertularia* highest growth rates were recorded at 15°C–20°C (Fig. 2B); however, at the highest light intensity growth rates of this strain did not differ significantly from 10°C to 25°C, i.e. the temperature effect was insignificant (one‐way ANOVA, *F*_3,8_ = 1.052, *P* = 0.421). At moderately low (40 μmol photon · m^−2^ · s^−1^) and very low (10 μmol photon · m^−2^ · s^−1^) light intensity, the growth rates of all three species declined with increasing temperature, except for *D. divergens* which was unaffected by temperature at the lowest light intensity tested.

**Table 2:** Three‐way ANOVA results for growth rates of *Dinobryon* spp. with *F*‐values and significance levels (*P*); df, factor degrees of freedom

Variable(s)	df	*F*	*P*
Temperature	3	47.31	<0.001
Species	2	15.37	<0.001
Light	2	593.56	<0.001
Temperature × species	6	17.74	<0.001
Temperature × light	6	17.52	<0.001
Species × light	4	7.78	<0.001
Temperature × species × light	12	5.05	<0.001

### Bacterial levels in experiments with *Dinobryon* spp

Bacterial abundance increased rapidly in all treatments at 10°C and 15°C, reaching 10^7^ cells · mL^−1^ 3 d after beginning of the experiments (Fig. S4 in the Supporting Information). There was no obvious difference in bacterial abundance between the three light levels and the two temperatures, nor between the three *Dinobryon* species. However, since we did not measure bacterial levels in all replicates and at the higher temperatures, we did not test if these trends were significant.

## Discussion

### Interactive effect of light and temperature on phytoplankton growth rates and temperature tolerance

We found a statistically significant light × temperature interaction, and this interaction was species‐specifically different within both *Cryptomonas* and *Dinobryon*, supporting our second and third hypotheses. As expected, reduced light intensity shifted the temperature at which specific growth rates were maximal (μ_max_) to lower temperatures (Edwards et al. 2016) in several species. For instance, in *Cryptomonas* sp. we observed a shift in μ_max_ from 25°C at 240 μmol μmol photon · m^−2^ · s^−1^ to 20°C at 200 μmol photon · m^−2^ · s^−1^ and 15°C at 50–100 μmol photon · m^−2^ · s^−1^ (Fig. 1, A and B). Similarly, *D. divergens* reached μ_max_ at 25°C when light intensity was 200 μmol photon · m^−2^ · s^−1^, but at 10°C–15°C when light intensity was 40 μmol photon · m^−2^ · s^−1^ (Fig. 2A). However, in *C. pyrenoidifera* (Fig. 1C) and *D. sociale* (Fig. 2C) the temperature at which μ_max_ was reached was invariant with or little affected by photon flux density. The meta‐analysis by Edwards et al. (2016) considered only the two *Cryptomonas* species investigated by Ojala (1993b; results reported in Table S3 in the Supporting Information) and no *Dinobryon* species. Therefore, the reasons why some mixotrophic (*Dinobryon*) and obligate phototrophic (*Cryptomonas*) species may not follow the general trend that *T*_opt_ for growth increases by ~4°C from (very) low irradiance (<20 μmol photon · m^−2^ · s^−1^) to ~100 μmol photon · m^−2^ · s^−1^ (Edwards et al. 2016) require further research.

Similar to many other phytoplankton taxa (reviewed by Edwards et al. 2016), lower light intensities reduced tolerance to high temperatures in *Cryptomonas* and *Dinobryon*. All species except *C. curvata* had positive growth rates over the entire temperature range when light intensity was high, but none of the species were able to grow at temperatures >20°C at the lowest light levels tested (i.e., 10 μmol photon · m^−2^ · s^−1^for *Dinobryon*, 50 μmol photon · m^−2^ · s^−1^ for *Cryptomonas*). The only seeming exception was *Cryptomonas* sp., which showed negative growth at 25°C but positive growth at 28°C when exposed to 40 μmol photon · m^−2^ · s^−1^ (Fig. 1A); however, its production rate was negative at 28°C (Fig. 1B). In agreement with our specific hypotheses, *Dinobryon* species were better able to cope with moderately low photon flux densities (40 μmol photon · m^−2^ · s^−1^) than *Cryptomonas* spp. (50 μmol photon · m^−2^ · s^−1^), in particular at higher temperatures (≥20°C).

### Species‐specific response to light and temperature in the genus *Cryptomonas*

Within the genus *Cryptomonas*, the largest species investigated, *C. curvata*, had the lowest growth and production rates and was most sensitive to temperatures exceeding 20°C. While the maximum growth rate obtained in this study is lower than its μ_max_ reported earlier (Table S3), *T*_opt_ and *T*_max_ are close to Ojala's results (Ojala 1993b). This author reported a lethal temperature for *Cryptomonas curvata* of 23.1°C, which is virtually identical to our observations. Ojala (1993b) used a different medium than the modified WC media used by Gervais (1997) and in the present investigation; therefore, it appears likely that differences in growth rates between studies reflect differences in media. However, since we used relatively wide temperature intervals of 3°C–5°C, our results reported in Table S3 allow only a crude estimate of *T*_opt_. With this restriction in mind, the experimentally determined *T*_opt_ (15°C–19°C; Ojala 1993b, this study) and *T*_max_ of ~23°C are consistent with field observations (reviewed by Reynolds 2006). For instance, in a Brazilian reservoir *C. curvata* was a dominant species during the austral winter when temperature ranged from 13°C to 18°C and declined during summer (Bicudo et al. 2009). Similarly, in many temperate lakes cryptophytes including *C. curvata* dominate in spring and early summer (reviewed by Reynolds 1984, Sommer 1986).

Taken together, the results summarized in Table S3 support the general conjecture that the larger cryptophytes (group Y in the functional classification of Reynolds et al. 2002) are tolerant to low light conditions and generally well‐adapted to live in a wide range of habitats. However, if the smaller *Cryptomonas* species (*Cryptomonas* sp. and *C. pyrenoidifera* in the present study) are included, it becomes obvious that tolerance to photon flux densities ≤50 μmol photon · m^−2^ · s^−1^ (low light) depends on temperature and generally decreases with increasing temperature. In contrast, tolerance to irradiances ≥100 μmol photon · m^−2^ · s^−1^ (high light, potentially causing photoinhibition) increases with temperature (Morgan and Kalff 1979) until *T*_opt_ is reached and then decreases (Cloern 1977, Edwards et al. 2016, this study).

### Species‐specific response to light and temperature in the genus *Dinobryon*

The specific growth rates that we measured for the three *Dinobryon* species were close to μ_max_ reported in the literature for the same and related species (Table S3). Similarly, *T*_opt_ for growth rates were close to earlier findings; our estimate of *T*_max_ in *D. sertularia* was higher than previously determined (Lehman 1976; Table S3).

In *Dinobryon*, species‐specific differences in the response to light and temperature could reflect differences in the rates of net photosynthesis and bacterial ingestion. Experiments with *D. sociale* showed that photosynthetic rates and bacterial ingestion were maximal at 16°C (Princiotta et al. 2016). Bacterial ingestion did not differ between high and medium light intensities (130 μmol photon · m^−2^ · s^−1^ vs. 66 μmol photon · m^−2^ · s^−1^, Princiotta et al. 2016), but increased when light was reduced to even lower levels s (25 μmol photon · m^−2^ · s^−1^; Heinze et al. 2013), suggesting that uptake of bacteria can support growth of *Dinobryon* when carbon acquisition from photosynthesis is low. In contrast, Jones and Rees (1994) found no effect of light on the ingestion rates of *Dinobryon divergens*, and Caron et al. (1993) reported higher ingestion rates at high than at low light intensity in *D. cyclindricum*. Measurements of ingestion rates in field samples detected higher bacterial ingestion rates in *D. divergens* than in *D. sociale* (Kamjunke et al. 2007). Collectively, these experiments indicate that species of *Dinobryon* might differ in their ability to supplement their nutrition with carbon acquired from uptake of bacteria, and such interspecific variation in ingestion rate could result in different tolerance of low light levels. In our study, *D. divergens* was more sensitive to low light than *D. sertularia* and *D. sociale* when temperatures were 10°C–15°C. At the upper end of the temperature gradient, however, *D. sociale* was more negatively affected by reduced light levels than the other two species. Quantifying variation in traits such as bacterial ingestion rate could be a useful approach for future experiments to elucidate reasons for interspecific variation in sensitivity to light and temperature.

Variation in bacterial abundances could have contributed to the observed treatment effects. However, bacterial abundance increased rapidly in our experiment, exceeding 10^7^ cells · mL^−1^ in all treatments 3 d after beginning of the experiments. Therefore, it is unlikely that food‐limitation was the reason for the negative growth rates recorded at low light levels.

### Response to high temperatures by *Cryptomonas* and *Dinobryon* – general implications

The major goal of this study was to investigate if sensitivity to high water temperatures (≥25°C) is a general characteristic of the genera *Cryptomonas* and *Dinobryon*. The answer is clearly negative, provided that the species are not nutrient or light limited. Under high nutrient and light levels, five of the six species that we investigated showed positive growth at 25°C (Figs. 1 and 2). The sensitivity of *Cryptomonas* and *Dinobryon* to high temperature observed in field studies (see Introduction) probably reflects interactive effects of temperature, light (including exposure to UV), and nutrients. Using similar experimental conditions as in the present study, Bergkemper et al. (2018) demonstrated that nutrient supply (i.e., soluble reactive phosphorus, SRP) significantly affects growth rates of *Cryptomonas* sp. strain SAG 26.80 and *Dinobryon* sp. (another unidentified *Dinobryon* species, isolated from Austrian Lake Lunz) and that this effect depends on the levels of temperature and light (light × temperature × nutrient interaction). In combination with nutrient stress, the experimental temperature of 26°C negatively affected growth rates of *Cryptomonas* sp. and *Dinobryon* sp. (Bergkemper et al. 2018). Interactions with competitors could be an additional reason for the sensitivity of *Cryptomonas* and *Dinobryon* to high temperature reported in field studies. Species can respond differently to temperature when grown in monoculture versus when grown within a community (Jiang and Morin 2004, Limberger et al. 2014), implying that competitive interactions can alter the response of species to warming. However, Weisse et al. (2016) reported that five algal species including *Cryptomonas* sp. strain 26.80 responded similarly to temperature and nutrient changes in P‐limited monocultures and multicultures.

Even if competition by other eukayotic phytoplankton may be of minor importance for *Cryptomonas*, competition for resources and other biotic interactions (e.g., predation, parasitism, mutualism) generally constrain the realized niche of phytoplankton; the latter represents often only a small subset of the environmental factors that can be potentially tolerated by an algal species in the absence of biotic interactions (reviewed by Karasiewicz et al. 2017, 2018). Similar considerations apply to the interactive effects of abiotic factors. The recent work conducted in our laboratory (Weisse et al. 2016, Bergkemper et al. 2018, this study) and elsewhere (reviewed by Edwards et al. 2016) demonstrated that interaction of temperature, light, and nutrients (i) significantly narrows the range of positive growth rates of common phytoplankton species and that (ii) variables such as temperature tolerance and light thresholds for growth, which may be used to predict the occurrence of species under altered environmental conditions, are context specific. In oligo‐mesotrophic Lake Mondsee, in which most of the species used in our study co‐occur, maximum SWT has increased by 6°C over the past one hundred years and now regularly exceeds 26°C in summer (Dokulil 2018). Since nutrients (i.e., soluble reactive phosphorus) are usually depleted in the epilimnion when SWT peaks (Bergkemper and Weisse 2017), we predict that most *Cryptomonas* and *Dinobryon* species will significantly decline in near‐surface waters if lake warming continues. However, flagellated algae might escape adverse conditions by migration to benign environments. *Cryptomonas* species perform phototactic responses to varying light conditions, conducting diurnal vertical migrations over several meters (Sommer 1985, Arvola et al. 1991, deNoyelles et al. 2016). This behavior may also serve to avoid temperatures above the species‐specific *T*_opt_ in situ (Bergkemper et al. 2018). Accordingly, in lakes with stable summer stratification and high surface temperatures (>20°C), several *Cryptomonas* species are dominant members of the algal assemblage causing the deep chlorophyll maximum (Pedrós‐Alió et al. 1987, Gervais 1998, Talling et al. 2005, deNoyelles et al. 2016), i.e., they peak under conditions of reduced light and temperature. Our results demonstrate that if water temperature exceeds 20°C, the niche of *Cryptomonas* species is constrained to light levels >50 μmol photon · m^−2^ · s^−1^. *Dinobryon* also exhibits migratory responses to temperature and light (Clegg et al. 2003a,b, 2007), and its critical light level at higher temperatures seems to be lower than in *Cryptomonas*.

Tolerance toward high water temperature (*T*_max_ in Table S3) is an important functional trait of phytoplankton (Bergkemper et al. 2018) that received relatively little attention traditionally but may become important in the course of global warming. Among the three *Cryptomonas* species that we investigated, *T*_max_ was negatively related to cell size. This is supported by Ojala's study (Ojala 1993a), who also found that the small cryptophyte *C. czosnowskii* tolerated higher maximum water temperature than the larger *C. curvata*. Since the data reported in Table S3 are confounded by different culture conditions used by the various investigators, it is not clear if this a general rule among *Cryptomonas* species. In addition, we observed declining cell sizes within species of *Cryptomonas*, indicating that populations might shift to smaller individuals. If these trends are general, they have important functional implications for the pelagic food web. Small cryptophytes (<1,000 μm^3^) are primarily ingested by small to medium‐sized ciliates and metazoan predators such as small rotifers and cladocerans (reviewed by Weisse 2006). Larger cryptophytes are more easily captured by large *Daphnia*, calanoid and cyclopoid copepods (Knisely and Geller 1986, Gliwicz 2003). In other words, small and large *Cryptomonas* species are not functionally equivalent, and the relative decline of large *Cryptomonas* at high water temperatures may shift the pelagic food web to the microbial food web.

In conclusion, the interactive effect of temperature with light and nutrients is an area that awaits further research in the course of lake warming. Similarly, the competitive abilities of *Cryptomonas* and *Dinobryon* in response to algal competitors and protist and metazoan predators need to be studied in more detail.

We thank Peter Stadler for technical assistance and gratefully acknowledge constructive comments provided by Thomas Pröschold and two anonymous reviewers. Funding was obtained from the University of Innsbruck, Austria.

## Supporting information


**Figure S1.** Cell volume of *Cryptomonas* sp. in the course of the experiments.

**Figure S2.** Cell volume of *C. pyrenoidifera* in the course of the experiments.

**Figure S3.** Cell volume of *C. curvata* in the course of the experiments.

**Figure S4.** Bacterial abundance in experiments with the three *Dinobryon* species at high (200 μmol photon · m^−2^ · s^−1^, top), moderately low (40 μmol photon · m^−2^ · s^−1^, middle) and very low (10 μmol photon · m^−2^ · s^−1^, bottom) photon flux densities and 10°C and 15°C. Standard deviation of the means is only reported at high light levels, because bacteria cell numbers were only measured in one replicate each at the lower light levels.

**Table S1.** Two‐way ANOVA results for growth and production rates of *Cryptomonas* spp. with *F*‐values and significance levels (*P*); df, factor degrees of freedom.

**Table S2.** Two‐way ANOVA results for growth rates of *Dinobryon* spp. with *F*‐values and significance levels (*P*); df, factor degrees of freedom.

**Table S3.** Maximum specific growth rates (μ_max_) of *Cryptomonas* spp. and *Dinobryon* spp. and their temperature optimum (*T*_opt_) and maximum temperature (*T*_max_) tolerated. L:D  =  Light:dark; LI = light intensity in μmol photon · m^−2^ · s^−1^; mod. = modified; MW = sterile filtered water from Lake Mondsee; mod. = modified; n.d. = not determined; Vol = cell volume. Species used in the present study are shaded.
