# Supplementary figures and images for: Temperature × light interaction and tolerance of high water temperature in the planktonic freshwater flagellates Cryptomonas (Cryptophyceae) and Dinobryon (Chrysophyceae)
Source: J Phycol. 2019 Jan 31;55(2):404–14. doi: 10.1111/jpy.12826 (PMC6590229; doi:10.1111/jpy.12826)

# *Cryptomonas* sp.

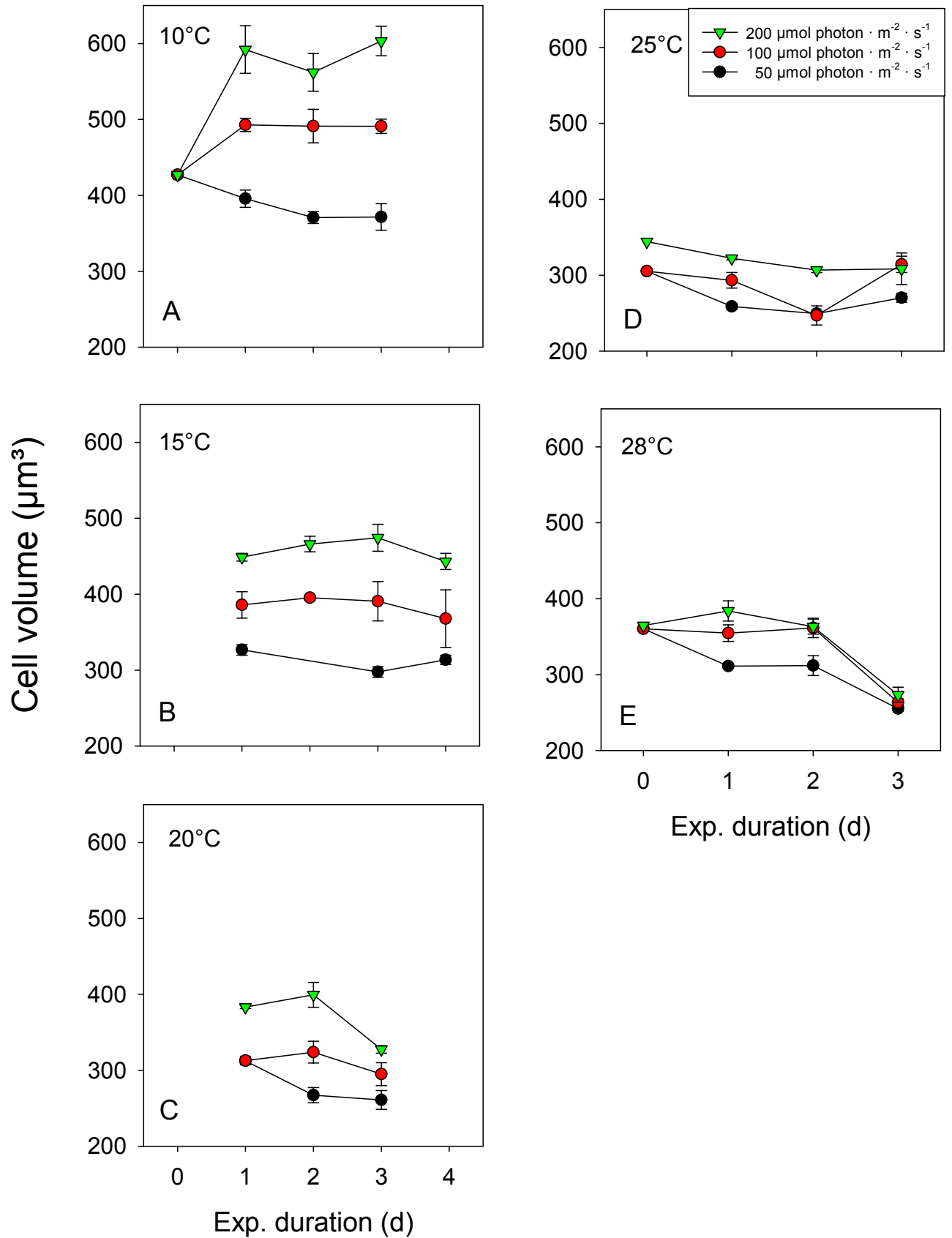

Fig. S1. Cell volume of *Cryptomonas* sp. in the course of the experiments.

Supplement: Supplementary file 1 — Figure S1. Cell volume of Cryptomonas sp. in the course of the experiments. [file JPY-55-404-s001.PDF]

# *Cryptomonas curvata*

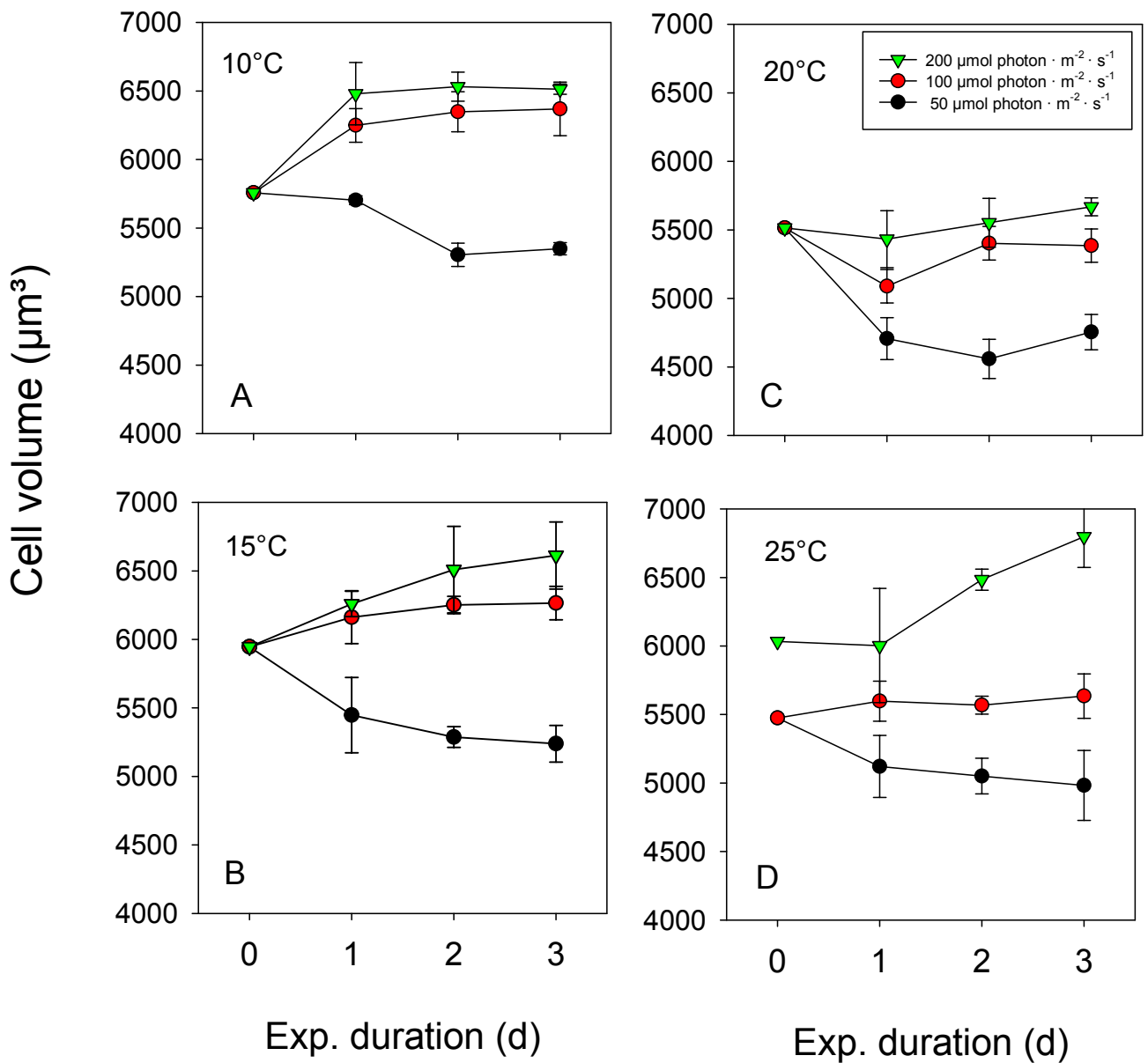

Fig. S3. Cell volume of *C. curvata* in the course of the experiments.

Supplement: Supplementary file 3 — Figure S3. Cell volume of C. curvata in the course of the experiments. [file JPY-55-404-s003.PDF]
