# Supplementary material for: Temperature × light interaction and tolerance of high water temperature in the planktonic freshwater flagellates Cryptomonas (Cryptophyceae) and Dinobryon (Chrysophyceae)
Source: J Phycol. 2019 Jan 31;55(2):404–14. doi: 10.1111/jpy.12826 (PMC6590229; doi:10.1111/jpy.12826)
Supplement: Supplementary file 2 — Figure S2. Cell volume of C. pyrenoidifera in the course of the experiments. [file JPY-55-404-s002.PDF]

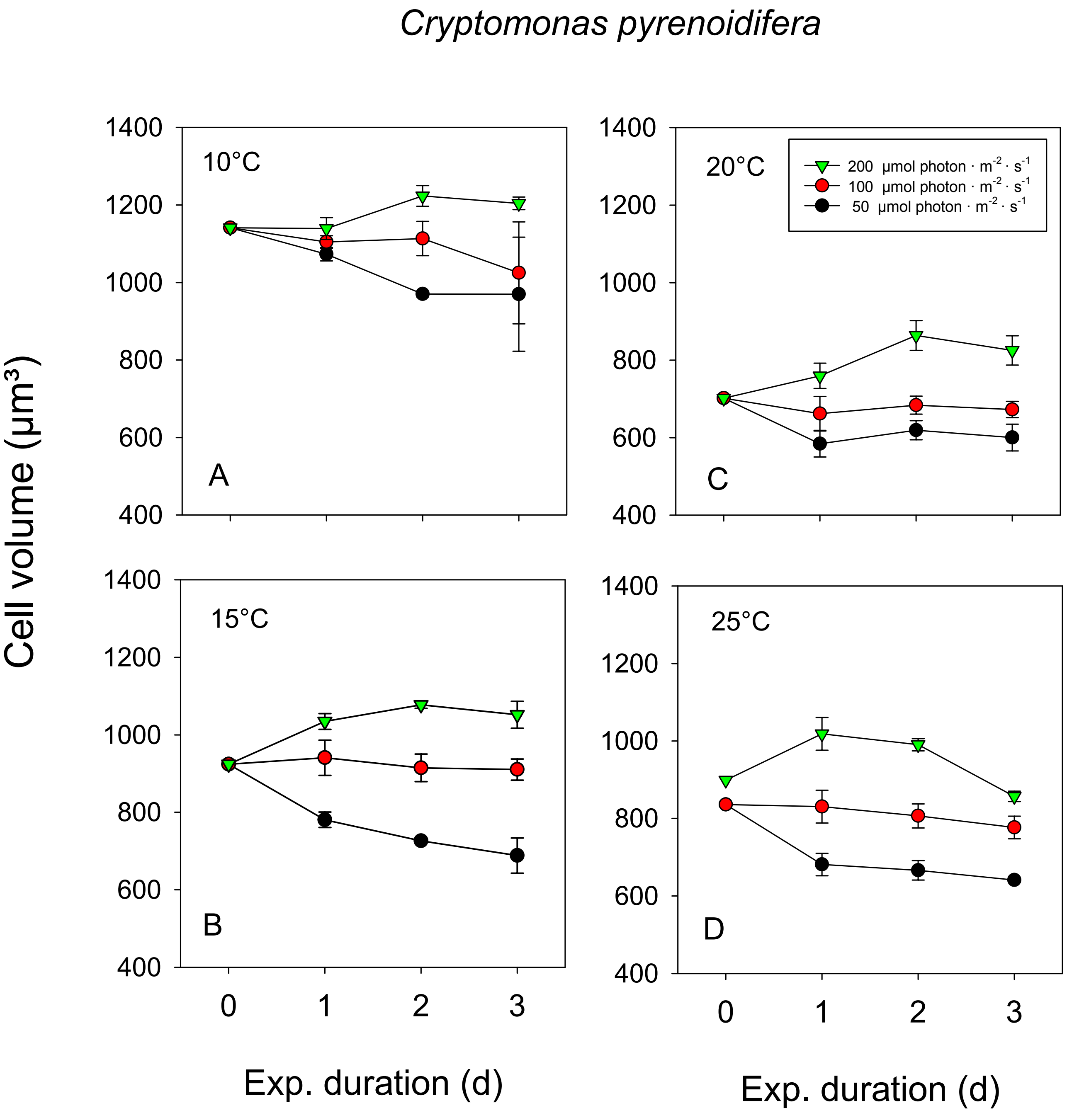

Fig. S2. Cell volume of *C. pyrenoidifera* in the course of the experiments.
