# Supplementary material for: Temperature × light interaction and tolerance of high water temperature in the planktonic freshwater flagellates Cryptomonas (Cryptophyceae) and Dinobryon (Chrysophyceae)
Source: J Phycol. 2019 Jan 31;55(2):404–14. doi: 10.1111/jpy.12826 (PMC6590229; doi:10.1111/jpy.12826)
Supplement: Supplementary file 4 — Figure S4. Bacterial abundance in experiments with the three Dinobryon species at high (200 μmol photon · m−2 · s−1, top), moderately low (40 μmol photon · m−2 · s−1, middle) and very low (10 μmol photon · m−2 · s−1, bottom) photon flux densities and 10°C and 15°C. Standard deviation of the means is only reported at high light levels, because bacteria cell numbers were only measured in one replicate each at the lower light levels. [file JPY-55-404-s004.PDF]

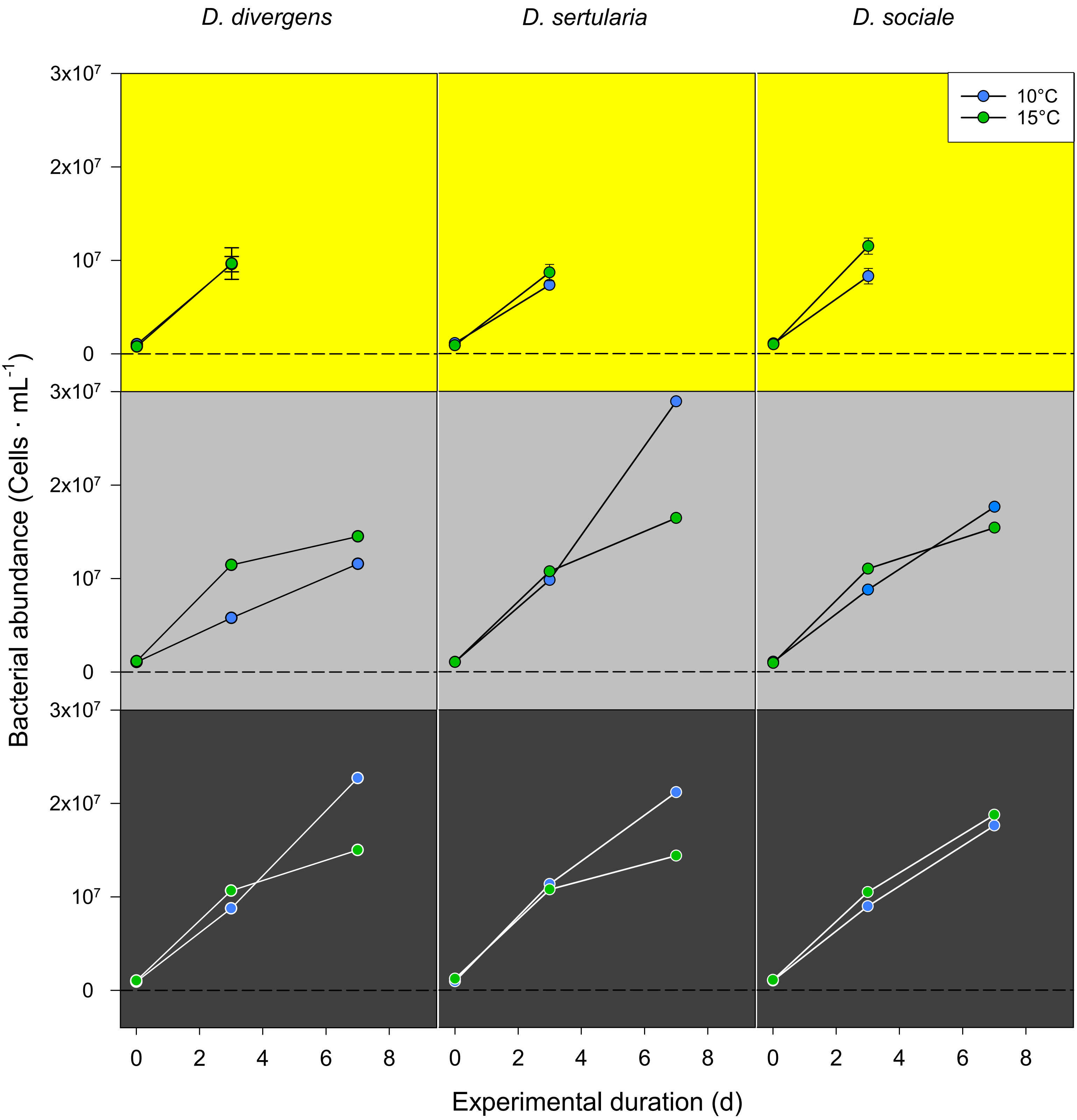

Fig S4. Bacterial abundance in experiments with the three *Dinobryon* species at high (200 µmol photon · m^{-2} · s^{-1}, top), moderately low (40 µmol photon · m^{-2} · s^{-1}, middle) and very low (10 µmol photon · m^{-2} · s^{-1}, bottom) photon flux densities and 10°C and 15°C. Standard deviation of the means is only reported at high light levels, because bacteria cell numbers were only measured in one replicate each at the lower light levels.
