# Supplementary material for: Temperature × light interaction and tolerance of high water temperature in the planktonic freshwater flagellates Cryptomonas (Cryptophyceae) and Dinobryon (Chrysophyceae)
Source: J Phycol. 2019 Jan 31;55(2):404–14. doi: 10.1111/jpy.12826 (PMC6590229; doi:10.1111/jpy.12826)
Supplement: Supplementary file 5 — Table S1. Two‐way ANOVA results for growth and production rates of Cryptomonas spp. with F‐values and significance levels (P); df, factor degrees of freedom. [file JPY-55-404-s005.docx]

Table S1. Two-way ANOVA results for growth and production rates of *Cryptomonas* spp. with F-values and significance levels (p); df= factor degrees of freedom

| Species | Variable(s) | df | | F | | p |
| --- | --- | --- | --- | --- | --- | --- |
| Growth rate (*µ*, d^-1^) |  |  | |  | |  |
| *Cryptomonas sp.* | Temperature | 4 | | 29.89 | | <0.001 |
|  | Light | 2 | | 535.7 | | <0.001 |
|  | Temperature × Light | 8 | | 40.34 | | <0.001 |
| *C. pyrenoidifera* | Temperature | 3 | | 60.83 | | <0.001 |
|  | Light | 2 | | 102.10 | | <0.001 |
|  | Temperature × Light | 6 | | 9.49 | | <0.001 |
| *C. curvata* | Temperature | 3 | | 180.83 | | <0.001 |
|  | Light | 2 | | 104.63 | | <0.001 |
|  | Temperature × Light | 6 | | 15.85 | | <0.001 |
| Production rate (*P*, d^-1^) |  | |  | |  |  |
| *Cryptomonas sp.* | Temperature | | 4 | | 11.60 | <0.001 |
|  | Light | | 2 | | 462.67 | <0.001 |
|  | Temperature × Light | | 8 | | 14.02 | <0.001 |
| *C. pyrenoidifera* | Temperature | | 3 | | 40.87 | <0.001 |
|  | Light | | 2 | | 81.29 | <0.001 |
|  | Temperature × Light | | 6 | | 6.85 | <0.001 |
| *C. curvata* | Temperature | | 3 | | 38.05 | <0.001 |
|  | Light | | 2 | | 29.98 | <0.001 |
|  | Temperature × Light | | 6 | | 3.17 | 0.019 |
