# Supplementary material for: Temperature × light interaction and tolerance of high water temperature in the planktonic freshwater flagellates Cryptomonas (Cryptophyceae) and Dinobryon (Chrysophyceae)
Source: J Phycol. 2019 Jan 31;55(2):404–14. doi: 10.1111/jpy.12826 (PMC6590229; doi:10.1111/jpy.12826)
Supplement: Supplementary file 6 — Table S2. Two‐way ANOVA results for growth rates of Dinobryon spp. with F‐values and significance levels (P); df, factor degrees of freedom. [file JPY-55-404-s006.docx]

Table S2. Two-way ANOVA results for growth rates of *Dinobryon* spp. with F-values and significance levels (p); df= factor degrees of freedom

| Species | Variable(s) | df | F | p |
| --- | --- | --- | --- | --- |
| *D. divergens* | Temperature | 3 | 3.13 | 0.045 |
|  | Light | 2 | 396.90 | <0.001 |
|  | Temperature × Light | 6 | 17.74 | <0.001 |
| *D. sertularia* | Temperature | 3 | 15.48 | <0.001 |
|  | Light | 2 | 130.60 | <0,001 |
|  | Temperature × Light | 6 | 4.44 | 0.004 |
| *D. sociale* | Temperature | 3 | 61.20 | <0.001 |
|  | Light | 2 | 226.93 | <0.001 |
|  | Temperature × Light | 6 | 12.99 | <0.001 |
