# Supplementary material for: Temperature × light interaction and tolerance of high water temperature in the planktonic freshwater flagellates Cryptomonas (Cryptophyceae) and Dinobryon (Chrysophyceae)
Source: J Phycol. 2019 Jan 31;55(2):404–14. doi: 10.1111/jpy.12826 (PMC6590229; doi:10.1111/jpy.12826)
Supplement: Supplementary file 7 — Table S3. Maximum specific growth rates (μmax) of Cryptomonas spp. and Dinobryon spp. and their temperature optimum (T opt) and maximum temperature (T max) tolerated. L:D = Light:dark; LI = light intensity in μmol photon · m−2 · s−1; mod. = modified; MW = sterile filtered water from Lake Mondsee; mod. = modified; n.d. = not determined; Vol = cell volume. Species used in the present study are shaded. [file JPY-55-404-s007.docx]

Table S3. Maximum specific growth rates (*µ_max_*) of *Cryptomonas* spp. and *Dinobryon* spp. and their temperature optimum (*T_opt_*) and maximum temperature (*T_max_*) tolerated . L:D=Light:dark; LI=light intensity in µmol photon m^-2^ s^-1^; mod. = modified; MW = sterile filtered water from Lake Mondsee; mod. = modified; n.d. = not determined; Vol=Cell volume. Species used in the present study are shaded.

| Species | Vol (µm³) | µ_max_ | Temperature/ Light conditions | Medium | *T_opt_* (°C) | *T_max_* (°C) | Reference |
| --- | --- | --- | --- | --- | --- | --- | --- |
| ***Cryptomonas* spp.** |  |  |  |  |  |  |  |
| *Cryptomonas* sp.^a^ | 170-540 | 0.40 | 16-21°C/ 16:8 L:D; LI=108 | mod. WC (CCAP) | n.d. | < 29 °C | Weisse et al., 2016 |
| *Cryptomonas* sp. ^a^ | ~280 | 0.36 | 9-24°C/ LI=70, constant light | mod. WC (CCAP) | 15 | 24-25 | Weisse et al., 2016 |
| *Cryptomonas* sp.^a^ | 320-880 | 0.60 | 15 °C/ LI=55, constant light | mod. WC (CCAP) | 20 | > 27 | Montagnes et al. 2008 |
| *Cryptomonas* sp. ^a^ | 260-605 | 0.59 | 20 °C/ 16:8 L:D; LI=200 | mod. WC (CCAP) | ~25 | > 28 | this study |
| *C. pyrenoidifera* (SAG 979-3) | 590-1,220 | 0.30 | 20 °C/ 16:8 h; LI=200 | Mod. WC (CCAP) | 20-25 | > 25 | this study |
| *C. curvata* ^b^ | 5000-9000 | 0.72 | 16-21 °C ^D^/ 16:8 h; LI ~115-195 | mod. Diatom | 19^j^ | 23.1 ^j^ | Ojala 1993 |
| *C. curvata* ^c^ | ~9,700 | 0.40 | 15 °C ^E^/ 13:11 L:D; LI=230 | mod. WC | n.d. | n.d. | Gervais 1997 |
| *C. curvata* ^b^ | 4,600-6,700 | 0.10 | 20 °C/ 16:8 h; LI ≈ 100 | Mod. WC (CCAP) | ~15 | 23 | this study |
| *C. czosnowskii* ^d^ | 500-900 | 1.39 | 26 °C/ 16:8 h; LI ~120 | mod. Diatom | 24.5 ^k^ | 30.4 ^k^ | Ojala 1993 |
| *C. erosa* | 400 - >9,000  (~1,400 at *T_opt_*) | 0.85 | 1-23.5 °C/ LI= 3-225, constant light | dilute *Cryptomonas* medium | ≥ 23.5 | > 23.5 | Morgan and Kalff 1979 |
| *C. marssonii* | n.d., ~250-920 ^i^ | 0.75-0.81 | 2-25 °C/ LI=100 µmol m^-2^ s^-1^, constant light | mod. Chu no. 10 | 11-17 | >25, <30 | Butterwick et al. 2005 |
| *C****.****.* cf. *ovata* | n.d., 1,100-1,520 ^i^ | 0.60 | 15 °C/ 13:11 L:D; LI=230 | mod. WC | n.d. | n.d. | Gervais 1997 |
| *C. ovata* var. *palustris* ^e^ |  | 0.58 | 20 °C/ 15:9 h; LI=150 | M3 | ~ 21-24 | > 26 | Cloern 1977 |
| *C. undulata* | ~3,800 ^j^ | 0.55 | 15 °C/ 13:11 L:D; LI=230 | mod. WC | n.d. | n.d. | Gervais 1997 |
| *C****.*** *phaseolus* | ~440 | 0.55 | 15 °C/ 13:11 L:D; LI=230 | mod. WC | n.d. | n.d. | Gervais 1997 |
| *Cryptomonas* sp.^f^ |  | 0.90 | 19 °C/ 550, constant light | f/2 |  |  | Sciandra et al. 2000 |
| ***Dinobryon* spp.** |  |  |  |  |  |  |  |
| *D. cylindricum* |  | ~0.7 | 8-24 °C/14:10 h; LI=64-128 | DyIII | 16-20 | >24 | Lehman 1976 |
| *D. divergens* |  | 0.72 | 2-30 °C/ LI=100, constant light | mod. Chu no. 10 | 17 | 25 - 30 | Butterwick et al. 2005 |
| *D. divergens* |  | 0.80 | 10-25 °C/ 16:8 h; LI=200 | DY-Vm + MW | ≥ 25 | > 25 | this study |
| *D. sertularia* |  | ~0.35 | 8-20 °C/ 14:10 h; LI=64-128 | DYIII | 16 | < 20 | Lehman 1976 |
| *D. sertularia* |  | 0.72 | 10-25 °C /16:8 h; LI=200 | DY-Vm + MW | 15-20 | > 25 | this study |
| *D. sociale* ^g^ |  | 0.31 | 16 °C/ 12:12 h; LI=67 | DY-IV | 16 | < 21 | Heinze et al. 2013 |
| *D. sociale* |  | ~0.7 | 14 °C?/ 14:10 h; LI=64-128 | DyIII | n.d. | n.d. | Lehman 1976 |
| *D. sociale* ^h^ |  | n.d. | 8-20 °C/14:10 h; LI=25-130 | DY-IV | 16 ^l^ | > 20 | Princiotta et al. 2016 |
| *D. sociale* |  | 0.65 | 10-25 °C /16:8 h; LI=10-200 | DY-Vm + MW | 10-20 | > 25 | this study |

^a^ SAG strain 26.80; ^b^CCAP strain 979/62; ^c^*C. rostratiformis* = *C. curvata* (Hoef-Emden et al. 2002, Hoef-Emden 2007, Choi et al. 2013)

^d^ CCAP strain 979/67; ^e^ UTEX strain 58; ^f^ unidentified species isolated from the Gulf of Naples; ^g^ UTEX strain 2267; ^h^ UTCC strain 392 species identification of i and h according to sequence analysis by R.A. Andersen (Princiotta et al. 2016); ^i^ according to Dokulil 1988; ^j^ cell volume recalculated from carbon content according to C (pg) = 0.216 V^0.939^ (Menden-Deuer and Lessard 2000); ^k^ model calculations based upon experimental data; ^l^ T_opt_ of photosynthesis

**References**

Butterwick, C., Heaney, S. I. & Talling, J. F. 2005. Diversity in the influence of temperature on the growth rates of freshwater algae, and its ecological relevance. *Freshwater Biol.* **50**:291-300.

Choi, B., Misun, K., Kim, J. I. & Shin, W. G. 2013. Taxonomy and phylogeny of the genus *Cryptomonas* (Cryptophyceae, Cryptophyta) from Korea. *Algae* **28**:307-30.

Dokulil, M. 1988. Seasonal and spatial distribution of cryptophycean species in the deep, stratifying, alpine lake Mondsee and their role in the food web. *Hydrobiologia* **161**:185-201.

Gervais, F. 1997. Light‐dependent growth, dark survival, and glucose uptake by cryptophytes isolated from a freshwater chemocline. *J. Phycol.* **33**:18-25.

Hoef-Emden, K. 2007. Revision of the Genus *Cryptomonas* (Cryptophyceae) II: Incongruences between the Classical Morphospecies Concept and Molecular Phylogeny in Smaller Pyrenoid-less Cells. *Phycologia* **46**:402-28.

Hoef-Emden, K., Marin, B. & Melkonian, M. 2002. Nuclear and Nucleomorph SSU rDNA Phylogeny in the Cryptophyta and the Evolution of Cryptophyte Diversity. *J Mol Evol* **55**:161-79.

Lehman, J. T. 1976. Ecological and nutritional studies on *Dinobryon* Ehrenb.: Seasonal periodicity and the phosphate toxicity problem. *Limnol. Oceanogr.* **21**:646-58.

Menden-Deuer, S. & Lessard, E. J. 2000. Carbon to volume relationships for dinoflagellates, diatoms, and other protist plankton. *Limnol.Oceanogr.* **45**:569-79.

Montagnes, D. J. S., Morgan, G., Bissinger, J. E., Atkinson, D. & Weisse, T. 2008. Short-term temperature change may impact freshwater carbon flux: a microbial perspective. *Glob. Change Biol.* **14**:2810-22.

Morgan, K. C. & Kalff, J. 1979. Effect of light and temperature interactions on growth of *Cryptomonas erosa* (Cryptophyceae). *J. Phycol.* **15**:127-34.

Ojala, A. 1993. Effects of temperature and irradiance on the growth of two freshwater photosynthetic cryptophytes. *J. Phycol.* **29**:278-84.

Princiotta, S. D., Smith, B. T. & Sanders, R. W. 2016. Temperature‐dependent phagotrophy and phototrophy in a mixotrophic chrysophyte. *J. Phycol.* **52**:432-40.

Sciandra, A., Lazzara, L., Claustre, H. & Babin, M. 2000. Responses of growth rate, pigment composition and optical properties of *Cryptomonas* sp. to light and nitrogen stresses. *Mar. Ecol. Prog. Ser.* **201**:107-20.
